# In vivo evaluation of the toxicity, genotoxicity, histopathological, and anti-inflammatory effects of the purified bioglycerol byproduct in biodiesel industry

**DOI:** 10.1186/s43141-020-00079-x

**Published:** 2020-10-15

**Authors:** Wesam Taha Basal, Aliaa Mahmoud Issa, Shehab Eldin Sayed Mohammed, Saher Abd-Elhafeez Mazen

**Affiliations:** 1 Department of Zoology, Faculty of Science, Cairo University, Cairo, Egypt; 2 Misr Petroleum Company Research Center, Cairo, Egypt; 3 Egyptian General Petroleum Corporation, Cairo, Egypt

**Keywords:** Acute toxicity, Anti-inflammatory, Bioglycerol, Carrageenan, Histopathology, Genotoxicity

## Abstract

**Background:**

Biodiesel has gained an increased popularity as a good alternative for fossil fuel because of its unusual qualities as a biodegradable, nontoxic, and renewable diesel fuel. Hence, the economic utilization of the accumulated bioglycerol byproduct became critically important for the sustainability of biodiesel industry. The purified bioglycerol might be used as a valuable industrial stock in cosmetic, medical, and food industries. However, if the purified product is going to be used in food, drug, or any industry that involves its ingestion or skin contact by human or animals, the product should be thoroughly tested on animal models.

**Results:**

The present study investigated the acute toxicity, anti-inflammatory, histopathological, and genotoxic effects of zeolite-purified biogylcerol on different animal models. All the previous tests proved the ability of the purification process to improve the qualities of the crude bioglycerol to a degree comparable to the pharmaceutical grade glycerol.

**Conclusion:**

In other words, it could be concluded that zeolite-purified bioglycerol can be used in different industries that involves products consumed by human or animals.

## Background

The increased risk of extinction of worldwide reserve of fossil fuels during the past few decades has aggravated the need for an alternative source of fuel due to environmental, socioeconomic and energy security concerns [1]. Biodiesel has already gained an increased popularity as a good alternative for fossil fuel because of its unusual qualities as a biodegradable, nontoxic, and renewable diesel fuel [2].

Bioglycerol is accumulated as a primary byproduct during the manufacture of biodiesel, accounting for about 20% of the total weight of the product [3, 4]. The sustainability of biodiesel industry requires the redirection and utilization of this huge amount of bioglycerol in another industries [5]. Unfortunately, bioglycerol is produced in a crude form that contains various impurities (organic and inorganic salts, soap, alcohol, traces of glycerides, and vegetable color) which limits its potential applications as a feedstock in different industries, hence lowering the chances of proper reuse [6–9]. On the other hand, the purified form is required as a valuable industrial feedstock especially in chemical industries; purified bioglycerol might have over than 2000 different applications, especially in the fields of food, cosmetics, and personal care products’ industries [10]. It might also be used in the manufacture of drugs, medicine, and pharmaceuticals [11, 12]. Several procedures are currently applied to purify crude bioglycerol including distillation, filtration, and adsorption (using activated zeolite) and ion-exchange [13]. In the zeolite method, the crude bioglycerol fraction is reacted with an acid (such as phosphoric acid) to convert the alkoxide salts and fatty acids to the corresponding alcohol and carboxylate salts, respectively. The resulting free fatty acid is removed by adsorption using zeolite or bleaching clay which offers easy, low operating cost and high efficiency method [12].

After the completion of purification process, standard physical and chemical properties (color, odor, water content, density, pH, free fatty acid content, and glycerol content) are measured to ensure that the purity has already reached above 90%. Degree of correlation between the purified bioglycerol and a standard pharmaceutical grade glycerol is usually confirmed via infrared (IR) spectroscopy [8].

If the purified product is going to be used in food, drug, or any industry that involves its ingestion by human or animals, the toxicity of the product should be thoroughly tested. Acute toxicity that results either from a single or multiple exposure was proved to be a very effective criterion to describe the toxic effects of a given substance in a short period of time [14]. Common signs of poisoning include unusual vocalization, tears, diarrhea, discharge, and bleeding from the eyes or mouth and convulsions [15, 16]. Mice and rats are always perfect choices as animal models for testing chemicals and drugs due to the similarities between their genome to that of the human, both containing 25,000–30,000 protein-coding genes [17, 18]. Moreover, approximately 80% of the rat genes have been reported to have orthologs in humans [19]. Most importantly, almost every human gene discovered to be associated with disease has an ortholog in the rat genome [20, 21]. Previous studies on oral acute toxicity revealed that the LD50 for glycerol was found to be more than 5 ml/kg in rats and mice where the oral lethal dose was 13–29 ml/kg and 15–38 ml/kg, respectively [22, 23]. Previous studies were also performed by Hashish et al. [24] and Hegazy et al. [25], in which they found that 25% of glycerol has a nephrotoxicity effect on rat models.

Inflammation is involved in the pathogenesis of many diseases and other life-threatening and debilitating disorders including aging, cancer, and cardiovascular dysfunction [26]. A well-known acute model of inflammation that is widely used for screening novel anti-inflammatory compounds in animals is the carrageenan-induced paw edema [27–29]. In a previous study, the properties of glycerol as an anti-inflammatory agent were tested by evaluation of ear swelling (by measuring thickness and weight) in 12-O-tetradecanoylphorbol-13-acetate-induced irritation in dehaired mice where the glycerol pretreatment prevented ear swelling in a non-dose-dependent manner [30]. In another review study, glycerol was used as a topical anti-inflammatory agent either solely or combined with other agents, in both cases; improvement in human dry skin was observed [31].

Along with its hydrating properties, glycerol was also found to prevent the phase transition of the stratum corneum (SC) lipids from liquid to solid crystalline structure, thus preventing water loss and improving skin barrier properties [32]. The extensive usage of glycerol as an indispensable component in almost all kinds of topical dermatological preparations implies a histopathological evaluation on the effect of purified bioglycerol on skin before using it as an ingredient in dermatological industries [30]. Histopathology is a valuable method for microscopic examination of *tissue* in order to study the manifestations of *disease* or experimenting a new drug or chemical [33, 34]. In a previous study, glycerol has been proved to be an effective skin-conditioning agent when incorporated into a lotion at concentration of 5% [35], which might be attributed to multiple positive effects on the physical properties of the skin, including increased hydration and improving elasticity. In another study, a formulation containing glycerol (5%) and xylitol (5%) was examined to study its effects on the hydration, barrier function, and morphological parameters of the skin [30]. In a recent study, a prepared chitosan-glycerol gel enhanced skin tissue regeneration and wound healing factors in acute full-thickness skin wounds in mouse animal models [36].

Genotoxicity is an important area of science which studies the genotoxic effects of a chemical agent to which the organism is exposed, using multiple available assays to assess the damage that these agents may cause to the DNA [37]. The use of alternative small animal models in both genotoxicity and toxicology has gained tremendous popularity in the last decade. *Drosophila melanogaster* was a premier model for developmental biologists and geneticists only, but its utility for toxicology studies has recently shown a wide spread emergence [38] Currently, *Drosophila* is being used in studies for a number of priority contaminants and toxicants [39]. In a previous study, Hifzur et al. [40] had shown that *Drosophila melanogaster* is a sensitive and suitable model for the in vivo assessment of genotoxicity using alkaline comet assay. The comet assay is being applied to *Drosophila melanogaster* since around 15 years ago and was known to be a very useful tool in genotoxicity and DNA repair testing [41]. It detects DNA strand breaks and alkali labile sites by measuring the migration of DNA from immobilized nuclear DNA [42]. Previous studies on the effect of glycerol on DNA integrity have shown some controversial results where the exposure of porcine blastocysts to glycerol and other cryoprotectants decreased their viability and increased the number of DNA-fragmented nuclei in the porcine embryos [43]. Another study gave opposite results where glycerol and other cryoprotectants increased nuclear DNA integrity in male mouse sperms [44].

## Objectives

The present study aims at determination of in vivo evaluation of the toxicity, genotoxicity, histopathological, and anti-inflammatory effects of the purified bioglycerol byproduct in biodiesel industry

## Methods

### Animal stock

All experiments and procedures were consented by the Institutional Animal Care and Use Committee (IACUC) with approval number (CUFS.S.Cell.Bio.35-15) from the ethical point of view and according to animal welfare act of the Ministry of Agriculture in Egypt that enforces the humane treatment of animals.

The Institutional Animal Care and Use Committee (IACUC) is organized and operated according to the World Organization for Animal Health (OIE) and Guide for the Care and Use of Laboratory Animals 8th Edition 2011.

#### Sprague Dawley rats’ strain

A total of 36 Sprague Dawley rats (18 males and 18 females) of 103–112 g were obtained from the animal house colony of the National Research Centre, Dokki, Giza, Egypt, and were kept separated in stainless steel cages at room temperatures of 25 ± 2 °C and a relative humidity of about 55%. Water and food were given ad libitum*.*

#### Swiss Albino mouse strain

A total of 30 Swiss Albino mice of 20–30 g body weight (15 males and 15 females) were obtained from the animal house colony of the National Research Centre, Dokki, Giza, Egypt. The animals were housed in standard metal cages in an air-conditioned room at 22 ± 3 °C, 55 ± 5% humidity, and provided with standard laboratory diet and water ad libitum.

#### *Drosophila melanogaster* strain (w^1118^ strain)

The used *Drosophila* strain w^1118^ was obtained from well-established colonies at the genetics department, Faculty of Agriculture, Ain-Shams University, Egypt. The strain was originated from Bloomington Drosophila stock center (stock no. 5905, FlyBase ID: FBst0005905).

### Chemicals

Bioglycerol was prepared by transesterification process as described by Cunha et al. [45] followed by purification using acidification process and filtration step using activated zeolite [8].

### Standard physical and chemical tests

Purified bioglycerol color and odor were manually examined. Cold oxidation of the bioglycerol was performed using sodium periodate in a strong acid medium; the produced formic acid was titrated with standard volumetric sodium hydroxide solution to determine the concentration and purity according to (ISO 2879-1975). Water content was determined by Karl Fischer titration technique according to (ASTM E203), and the density was measured according to (ASTM D4052) specification. Free fatty acid (FFA) content was measured by titration according to (ASTM D1980), and pH was determined using pH meter. The chemical bonding of purified bioglycerol was verified and compared to that of the pharmaceutical glycerol using Fourier Transform Infrared Spectroscopy (FTIR), model BRUKER Alpha II. The light source was adjusted to the middle range infrared (4000–500 cm^−1^) and 4 cm^−1^ resolution according to (ASTM E1252 – 98) specifications.

### Acute oral toxicity study

Twenty-four rats were randomly divided into 2 groups of 12 rats each (6 males and 6 females). Limit acute toxicity test was performed on the two groups; control group received 5 ml/kg of distilled water, while treated group received 5 ml/kg of zeolite-purified bioglycerol orally administered by stomach tube [46]. Rats were observed for 14 days for any signs of acute toxicity and changes in the skin, fur’s color, and texture, respiratory, circulatory system, and abnormal somato-motor activity or behavior pattern during the propagation of test. Changes in specific organs’ weight (liver, kidney and heart) were recorded as means and standard deviations [47].

### Anti-inflammatory and acute inflammation (carrageenan-induced paw edema method)

Twelve rats were randomly divided into two groups of 6 rats each (3 males and 3 females). The left hind paw of the rats in the treated group was topically treated with the purified bioglycerol (5%), while paws of those in the control group were treated with 0.9% NaCl solution under the same experimental conditions [48]. One hour after the application of the tested materials, each rat received a 0.1-ml subplanter injection of 1% carrageenan suspension (Sigma, USA) in the same paw. The thickness of the paw of each rat was measured at 1-, 2-, 3-, and 4-h intervals after the injection of the inflammatory agent. The degree of inflammation was calculated by subtracting the thickness of foot before inflammation from that of the inflamed foot at the different times intervals, and then the inhibition effect of the bioglycerol was calculated according to Lanhers et al. [48] by the following equation:

$$ \mathrm{Inhibition}\%=\frac{\mathrm{control}\ \mathrm{inflamed}\ \mathrm{area}\ \mathrm{thickness}\ \mathrm{mean}-\mathrm{sample}\ \mathrm{inflamed}\ \mathrm{area}\ \mathrm{thickness}\ \mathrm{mean}}{\mathrm{Control}\ \mathrm{mean}}\times 100 $$

### Histopathological study on the skin

Thirty mice were randomly divided into 5 groups, each containing 6 individuals, 3 males and 3 females as follows: unpurified bioglycerol group, partially purified bioglycerol group (after 1st zeolite’s adsorption), completely purified bioglycerol group (after 3rd zeolite adsorption), pharmaceutical glycerol group, and a control group (sterile distilled water). The hair in an area of approximately 3 × 2 cm on the abdomen was trimmed, using scissors, carefully not to damage the skin. Mice with any visible sign of skin damage were excluded. Glycerol and bioglycerol used in this treatment were prepared as 5% solution in a volume of 1 ml, then applied to the exposed abdominal skin, and were spread evenly over the entire area. This was repeated once daily for 2 weeks; then, the animals were sacrificed by decapitation at the end of the 14th day. Treated skin areas from all animals were immediately removed and fixed for 72 h in 10% neutral-buffered formalin. They were then washed under running tap water for half an hour, dehydrated, cleared, and embedded in paraffin [49]. Serial sections of 6 μm thick were cut using microtome (PFM Medical ^TM^) and stained with hematoxylin and eosin (Sigma-Aldrich) for histopathological investigation [50].

### Assessment of DNA fragmentation by comet assay (single cell gel electrophoresis, SCGE)

All crosses of normal and treated *Drosophila* were reared at 25 °C on a standard *Drosophila* rearing medium [51]. The mixture was poured into rearing bottles (12 cm height × 6 cm diameter) and left to solidify at 25 °C to an experienced consistency suitable for rearing. Parental flies of the isogenic strain w^1118^were checked for any phenotypic abnormalities. Only clean flies were used for oviposition. The hatched larvae were left to grow and develop to the 3rd instar and collected after 96 h post oviposition (72 h after hatching) by floating over 20% glycerol solution. The collected larvae were briefly washed with tap water, dried on filter paper and transferred into five groups of new rearing bottles where the rearing medium was mixed with the tested compounds; distilled water as a control sample, 5% of each: unpurified bioglycerol, partially purified bioglycerol, completely purified bioglycerol, and pharmaceutical grade glycerol. The larvae were left to feed on the treated medium for 24 h. The treated larvae were floated from the treatment medium with 20% glycerol, briefly rinsed with tap water, dried on filter paper, and transferred to a new bottle containing a normal rearing medium (no addition included) until pupation and adult emergence. Extent of DNA strand breaks in all types of cells of *Drosophila* adult were then assessed using the alkaline comet assay [52]. Adult flies were frozen in liquid nitrogen, around 100 flies were gently homogenized into powder, and then an alkaline comet assay was performed [53].

## Results

### Standard physical and chemical tests

**Fig. 1:**
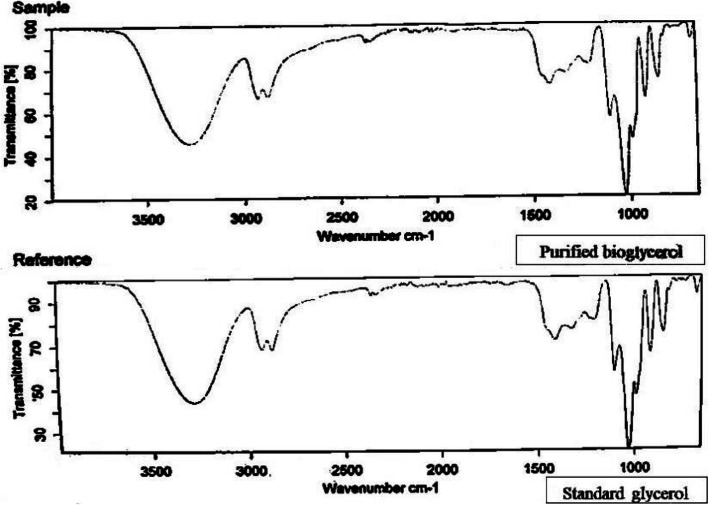
FTIR spectrum for both (standard or pharmaceutical grade glycerol and purified bioglycerol) where the correlation between two samples is (99.84 %)

The physical and chemical properties of the purified bioglycerol—odor, color, water content, density, and pH—were compared to both the unpurified form and the pharmaceutical grade glycerol. Unlike the unpurified bioglycerol, after purification the bioglycerol properties were highly similar to that of the pharmaceutical glycerol (Table 1). A Fourier-transform infrared spectrum of pharmaceutical grade glycerol was almost identical to that of the purified bioglycerol with a correlation up to 99.84%. The FTIR spectrum of the purified bioglycerol showed the absence of peaks at 1580 cm^−1^and 1740 cm^−1^ indicating the complete removal of impurities like free fatty acid and methyl ester compounds (Fig. 1).

**Table 1:** Comparison between some chemical and physical properties of the unpurified bioglycerol, purified bioglycerol by activated zeolite, and standard or pharmaceutical grade glycerol

Tests	Unpurified bioglycerol	Purified bioglycerol by activated zeolite	Standard or Pharmaceutical grade glycerol
**Odor**	**Fatty odor**	**Odorless**	**Odorless**
**Color and appearance**	**Brownish black**	**Transparent**	**Transparent**
**Glycerol %**	**65.42**	**99.21**	**99.92**
**Water %**	**13**	**0.29**	**0.28**
**Density %**	**1.24**	**1.26**	**1.26**
**pH**	**11.21**	**6.96**	**7.01**
**Free fatty acid%**	**8.53**	**0.78**	**0.05**

### Acute oral toxicity test

Glycerol is generally classified as safe by the FDA and is extensively used in food and drug industries as a humectant or solvent. The safety of oral ingestion of the purified bioglycerol was tested by limit toxicity test in Sprague Dawley rats. No mortality or signs of deleterious effects were recorded at the tested dose (5 ml/kg) till the completion of the experiment period. No physical changes—tremors, convulsions, salivation, or lethargy—were detected throughout the testing period. Animals in the bioglycerol-treated group had shown a slightly change in the mean organs weight compared to the control group. In conclusion, the current study has proved the safety of the purified bioglycerol by revealing neither deaths nor toxic side effects or impactful big changes in the targeted organs weight with the tested dose (5 ml/kg) during a study period of 14 days (Table 2).

**Table 2:** Effect of 5 ml/kg dose of purified bioglycerol on some organs weight in male and female Sprague Dawley rats. Number of used rats (*n* = 24)

Parameters	Control (distilled water)		Bioglycerol dose (5 ml/kg)	
	Male	Female	Male	Female
**Mean Liver weight (g)**	**2.20**^**a**^ **± 0.20**	**2.24**^**a**^ **± 0.41**	**2.25**^**b**^ **± 0.46**	**2.29**^**b**^ **± 0.54**
**Mean Kidney weight (g)**	**0.33**^**a**^ **± 0.05**	**0.31**^**a**^ **± 0.06**	**0.39**^**b**^ **± 0.03**	**0.35**^**b**^ **± 0.05**
**Mean Heart weight (g)**	**0.30**^**a**^ **± 0.06**	**0.31**^**a**^ **± 0.06**	**0.31**^**b**^ **± 0.02**	**0.33**^**b**^ **± 0.02**

In each horizontal row and same sex, means with different letters are significantly different (*p* < 0.05)

### Anti-inflammatory effect and acute inflammation study

The carrageenan-induced paw edema method was proved to be one of the most feasible methods to screen anti-inflammatory agents. In a previous study, glycerol pretreatment prevented ear swelling in a non-dose dependent manner. Hence, in the present study, the anti-inflammatory effect of one dose of the purified bioglycerol (5%) was tested on carrageenan-induced edema as an inflammatory agent. The results of the current study clearly showed significant anti-inflammatory properties of the tested bioglycerol (5%) and established the ability of the purified bioglycerol to inhibit the carrageenan-induced rat paw edema and reduce the mean paw volume starting from the end of the second hour by 7% to the end of fourth hour by 35% (Table 5). The anti-inflammatory action of the bioglycerol started against acute inflammation of carrageenan by inhibiting some of the mediators released after carrageenan injection till the end of 4th hour. It could be concluded that the purified bioglycerol at the used concentration is effective in inducing an anti-inflammatory activity by reducing the acute inflammation response by 35% compared to control (Tables 3, 4, and 5).

**Table 3:** Thickness of the inflamed area in hind paws of control group (mm) *(n = 6)*

Rat no.	Time (h)			
	1	2	3	4
	The thickness of the inflamed area in hind paws (mm)			
**1**	**0.10**	**0.15**	**0.15**	**0.20**
**2**	**0.10**	**0.10**	**0.10**	**0.15**
**3**	**0.05**	**0.10**	**0.20**	**0.20**
**4**	**0.10**	**0.15**	**0.20**	**0.20**
**5**	**0.15**	**0.20**	**0.25**	**0.25**
**6**	**0.15**	**0.15**	**0.20**	**0.20**
**Mean**	**0.11**	**0.14**	**0.18**	**0.20**
**SD**	**0.04**	**0.04**	**0.05**	**0.03**
**SE**	**0.02**	**0.02**	**0.02**	**0.01**

**Table 4:** Thickness of the inflamed area in hind paws of the bioglycerol-treated group (mm) *(n = 6)*

Rat no.	Time (h)			
	1	2	3	4
	The thickness of the inflamed area in hind paws (mm)			
**1**	**0.10**	**0.10**	**0.15**	**0.20**
**2**	**0.10**	**0.20**	**0.10**	**0.10**
**3**	**0.07**	**0.15**	**0.15**	**0.20**
**4**	**0.10**	**0.15**	**0.15**	**0.15**
**5**	**0.15**	**0.15**	**0.15**	**0.05**
**6**	**0.14**	**0.05**	**0.15**	**0.05**
**Mean**	**0.11**	**0.13**	**0.14**	**0.13**
**SD**	**0.03**	**0.05**	**0.02**	**0.06**
**SE**	**0.01**	**0.02**	**0.01**	**0.03**

**Table 5:** The mean of anti-inflammatory activity of the purified bioglycerol sample on carrageenan-induced rat hind paw

Treatment	Time (h)			
	1	2	3	4
	The mean thickness of the inflamed area in hind paws (mm)			
**Control**	**0.11ª ± 0.02**	**0.14ª ± 0.02**	**0.18ª ± 0.02**	**0.20ª ± 0.01**
**Bioglycerol 5%**	**0.11ª ± 0.03**	**0.13**^**b**^ **± 0.02**	**0.14**^**b**^ **± 0.03**	**0.13**^**b**^ **± 0.04**
**Inhibition %**	**0**	**7**	**22**	**35**

The statistical analysis was done using one-way ANOVA to compare groups at the different time intervals. Data is expressed as mean values ± standard error. In each column, means with different letters are significantly different (*p* < 0.05)**.**

### Skin histopathological study

**Fig. 2:**
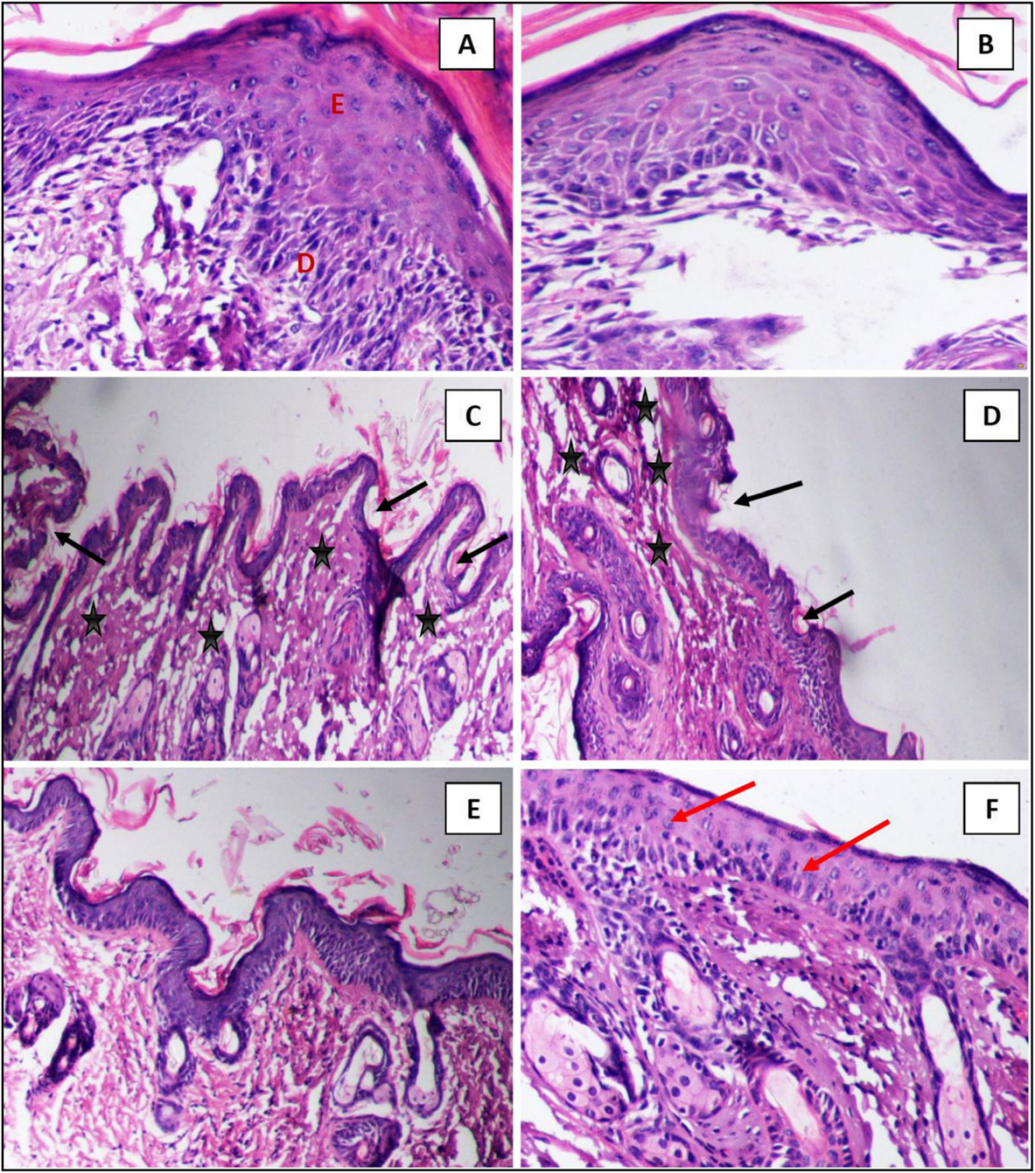
Photomicrograph of vertical sections in skin of all tested groups. **a** Normalappearance in both epidermis E and dermis D in control group. **b** Notable increase in thickness of epidermis E in pharmaceutical glycerol (5%) group. **c**, **d** Ulcers (black arrow) and shrinkage in the epidermis layer and degeneration of dermis (stars) was noticeable in the unpurified bioglycerol group (5%) (black stars). **e** Slight shrinkage and irregularity of epidermal layer accompanied by normal appearance of dermis in partially purified bioglycerol group (5%) (after 1st zeolite's adsorption). **f** Notable increase in thickness of epidermis and dermis of purified bioglycerol group (5%) (red arrow)

A histopathological study of skin sections treated with sterile distilled water, pharmaceutical glycerol, unpurified bioglycerol, partially purified bioglycerol, and purified bioglycerol was employed to detect any changes in the skin layers during a period of 14 days experiment (Fig. 2). The control group skin sections showed a normal histological appearance exhibiting a normal intact stratified squamous epidermis and dermis layer with abundant capillaries and connective tissue cells. In the unpurified bioglycerol group, the treated skin area became extensively dried starting the second day of treatment with the appearance of multiple ulcerated spots on the treated area that can be easily detected by naked eye. Upon examination under light microscope, a severe shrinkage, erosion, and cellular degeneration in some spots of the epidermis accompanied by degeneration of dermis were detected. In the skin sections collected from partially purified bioglycerol group, there was a slight shrinkage of epidermis in some spots and a normal appearance in others, while the dermal layer appeared totally unaffected. An increase in thickness of the epidermal and the dermal layers was observed in the skin sections of both pharmaceutical glycerol (5%) and purified bioglycerol (5%) groups. They penetrated the skin and created a “reservoir” in the depth of stratum corneum SC, which is the outermost layer of the epidermis, within the lipid bilayers without disruption of liquid crystallinity or lamellar structure. They also caused an obvious intracellular expansion of the corneocytes and intercellular expansion between corneocytes and are suggested to enhance skin barrier properties to improve the water-holding capacity of SC resulting in more moisturization of the skin. The observed results backed up the safety of zeolite purified bioglycerol and proved that its skin care quality can be compared to the pharmaceutical glycerol. It also proved the efficacy of the purification process that vanished the harmful effects realized with the unpurified bioglycerol.

### Single-cell gel electrophoresis (comet assay)

**Fig. 3:**
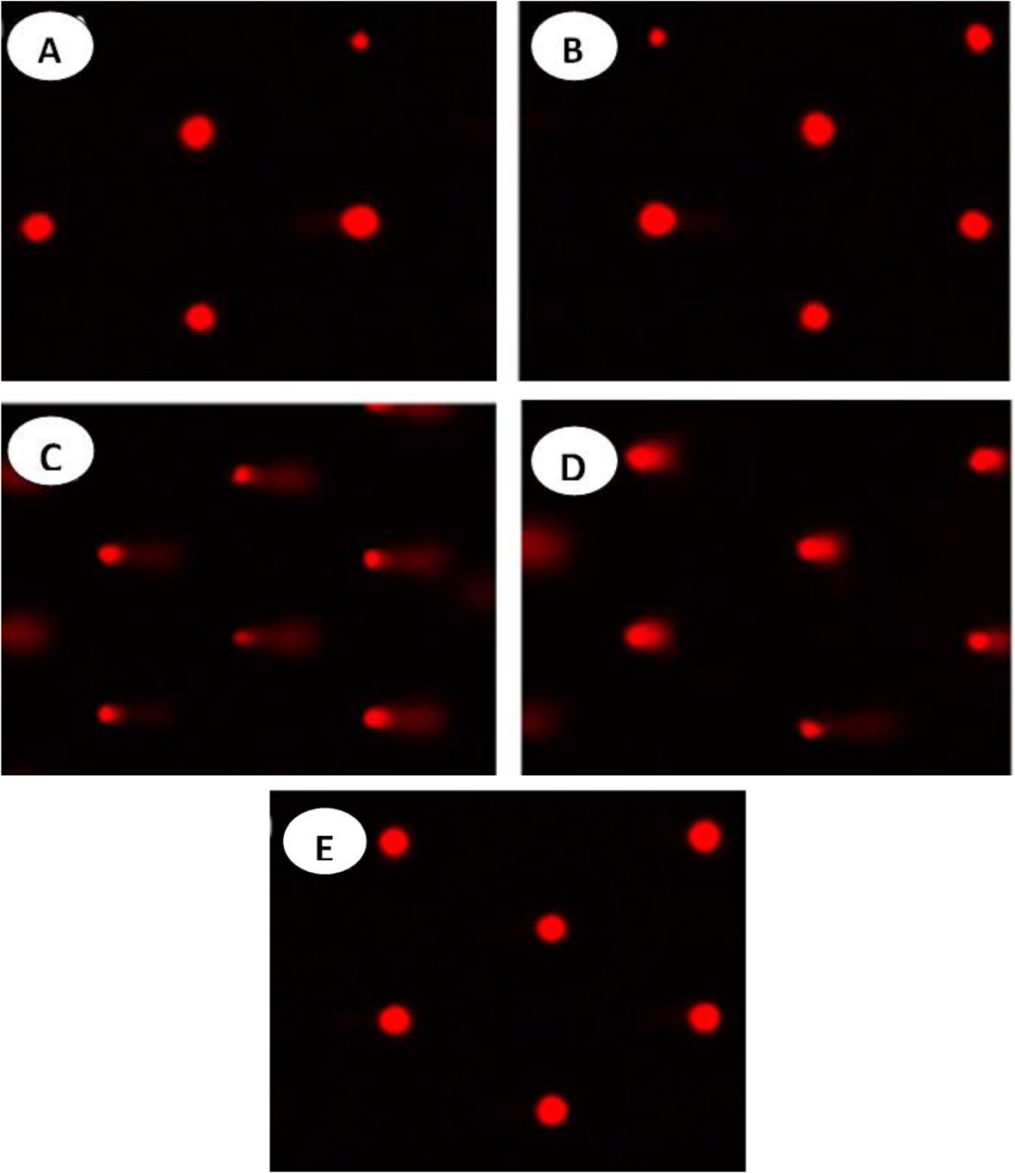
Comet assay images representing DNA damage in adult *Drosophila* whole body cells: **a** DNA damage in adult *Drosophila* whole body cells of isogenic strain of Drosophila (w1118) exposed to sterile distilled water (control), **b** pharmaceutical glycerol (5%), **c** unpurified bioglycerol (5%), **d** partially purified bioglycerol (5%)(after 1st zeolite’s adsorption), and **e** purified bioglycerol (5%)

DNA damage in cells was detected using a microgel electrophoresis technique known as single-cell gel electrophoresis or the comet assay. The quantitative estimation of the DNA damage in the whole-body cells of the control and treated parental adult flies of the isogenic strain (w^1118^) of *Drosophila* was accomplished by the single-cell gel electrophoresis under a strong alkaline condition (pH < 13) (Fig. 3).

The obtained data included the tail length (μm), % DNA concentration in the tail, and the calculated tail moment (Table 6). Tail moment values for DNA of flies treated with purified bioglycerol (5%) and pharmaceutical glycerol (5%) were very close and have no significant difference with the tail moment values obtained from the DNA of the control flies. On the other hand, the tail moment values of the DNA of partially purified bioglycerol (5%)-treated flies showed a slightly significant difference compared to the control group. The calculated tail moment value of the DNA of unpurified bioglycerol (5%)-treated flies exhibited a highly significant difference when compared to control group. These results proved the efficiency of the purification method, as the genotoxicity of the bioglycerol was decreased with each purification step rendering the purified bioglycerol safe to use.

**Table 6:** Quantitative estimation of the DNA damage by the comet assay, expressed as tail moment and tail length in whole body cells of parental adult of an isogenic strain (w^1118^) of *Drosophila*

Group	Tailed %	Untailed %	Tails length μm	Tail DNA%	Tail moment
**Distilled water (xontrol)**	1.5	98.5	**1.32 ± 0.12**	1.48	**1.95**
**Pharmaceutical glycerol (5%)**	2.5	97.5	**2.02 ± 0.26**	1.81	**3.66**
**Unpurified glycerol (5%)**	13	87	5.7 ± 0.19*	4.09	**23.31**
**Partially purified bioglycerol (after 1st zeolite’s adsorption) (5%)**	4.8	95.2	**3.63 ± 0.19**	2.57	**9.33**
**Purified bioglycerol (5%)**	2.7	97.3	**2.16 ± 0.16**	1.88	**4.06**

*Highly significant difference from the control group (*P* < 0.05)

## Discussion

### Standard physical and chemical tests

In a previous study, an FTIR analysis compared between an unpurified bioglycerol, purified bioglycerol, and a pharmaceutical grade glycerol to determine the presence or absence of different functional groups. The results showed the presence of peaks (1580 cm^−1^and 1740 cm^−1^) in the unpurified bioglycerol and the absence of those peaks in both purified bioglycerol and pharmaceutical grade glycerol [54]. The absorbance peak at 1580 cm^−1^ indicates the presence of impurities containing carboxylate ions (COO-), and the peak at 1740 cm^−1^ indicates the presence of carbonyl group (C=O) of an ester or carboxylic acids [54].

### Acute oral toxicity test

Most of the orally administered glycerol (nearly 80%) occurs in the liver as binding with free fatty acids to form triglycerides (lipogenesis) that can be distributed to adipose tissues [55]. Glycerol could also be incorporated into body fats, metabolized by glycerokinase to water and carbon dioxide, or it is utilized in glucose or glycogen synthesis, and approximately the rest 10–20% of the administered glycerol is utilized in the kidney [55]. This might be the reason for absence of statistically significant differences in the organs’ weight between rats of the control group treated with distilled water and those of the bioglycerol treated group. Glycerol in general is not toxic after ingestion, but it becomes toxic at high doses which are usually between 70 and 105 g [56].

### Anti-inflammatory effect and acute inflammation study

Zaman et al. [57] proved the ability of glycerol to reduce blood flow velocity and vessel diameter of micro-vasculature and decrease dermal blood flow and other indicators of inflammation. In another study, 5% glycerol was proved to have an anti-irritant and anti-inflammatory effects; it stated in the same study that it was able to prevent the inflammation related-increase in blood flow by reducing the accumulation of neutrophil, granulocytes, and lymphocytes and moderating the expression of inflammatory cytokines [58]. The anti-inflammatory effect of the bioglycerol might be explained by the same mechanism suggested for the anti-inflammatory action of glycerol in a previous study by the inhibition of cornerstone mediators of inflammatory response, and this happens when keratinocytes produced cytokines and chemokines (e.g., IL-1ɑ, IL-1β, TNF-ɑ, and IL-8) after the inflammation charge, the cytokines began activating other cell types (e.g., dendritic, Langerhans, and endothelial cells) whose cytokine release neutrophil granulocytes, macrophages, lymphocytes, and mast cells [59]. Both cytokine proteins, TNF-ɑ (tumor necrotic factor alfa) and IL-1β (Inrerleukin-1 beta), are responsible for migration of dendritic and Langerhans cells then activation of these cells respectively [60]. Glycerol was able to decrease the m-RNA expression of the inflammatory mediators IL-1β and TNF-ɑ in order to stop the progress of the irritant mechanism and prevented the continuation of the acute inflammation [58].

### Skin histopathological study

The hygroscopic properties of glycerol made it an indispensable component in cosmetics industries especially skin care products and dermatological preparations which work effectively against aging and various dermatological disorders that are accompanied by dry skin [30]. The epidermis was the most affected skin layer by unpurified bioglycerol; epidermal ulcers were previously attributed to segmental or more extensive loss of the epidermal layers including the basement membrane [61–64]. Exposure to the concentrated potassium hydroxide and methyl alcohol present as impurities in the crude bioglycerol might be the main reason for the appearance of ulcers and some erosion blots. This could be a good explanation for the thinning and the shrinkage which happened to the treated epidermal layer. In the partially purified bioglycerol group, the slight shrinkage of epidermis in some spots and normal appearance in others in addition to the unaffected dermal layer might be attributed to the partially increased purity of the incomplete purified bioglycerol. The improved appearance of the skin layers compared to the unpurified glycerol might also be explained by the beginning of pulling up water from the deep skin layers towards the epidermal layer resulting in hydration. The increase in the epidermal and the dermal thickness observed in both pharmaceutical glycerol (5%) and the purified bioglycerol (5%) groups could be due to the great capability of the glycerol in moisturizing the skin layers particularly the epidermal layer by attracting and binding the water from the deep epidermal layers and effectively hydrating the skin [30]. It should be noted that the glycerol is one of the most effective moisturizers that strongly hydrate the SC [30]. It diffuses and highly accumulates in the entire thickness of the SC [35, 65–67]. Glycerol was previously reported to have the ability of causing intracellular expansion of the corneocytes and intercellular expansion between corneocytes and is suggested to enhance skin barrier properties to improve the water-holding capacity of SC, an effect named “bulking” [65, 68].

### Single-cell gel electrophoresis (comet assay)

In consistence with the results obtained in the present study, pharmaceutical grade glycerol was proved in a previous work to be non-genotoxic compound in multiple Ames tests using multiple strains of *Salmonella typhimurium* at concentrations up to 50 mg/plate, cytogenetic assay, X-linked HGPRT, sister chromatid exchange assay, unscheduled DNA synthesis assay, and chromosome aberration test at concentrations up to 1.0 mg/ml [69]. The deleterious effect of the unpurified bioglycerol on the DNA might be attributed to the presence of some remnants of phosphoric acid and alcohol which also explains why this effect was totally diminished after the purification process.

## Conclusions

Unpurified bioglycerol cannot be used directly in any of the industries that involve human or animal consumed products. The present study proved that the unpurified bioglycerol has a significant destructive effect on skin tissue and genotoxic effect on DNA. On the other hand, zeolite-purified bioglycerol had shown almost similar characteristics to pharmaceutical grade glycerol with fair anti-inflammatory effect. The skin destructive and genotoxic effects of the unpurified bioglycerol were greatly diminished by zeolite purification process. It can be safely concluded that zeolite-purified bioglycerol is suitable for use in products consumed by human or animals.

## Data Availability

All data generated or analyzed during this study are included in this manuscript.

## Abbreviations

DNA: Deoxyribonucleic acid
IACUC: The Institutional Animal Care and Use Committee
ISO: International organization for standardization
ASTM: American society for testing and materials
FFA: Free fatty acid
FTIR: Fourier-transform infrared spectroscopy
FDA: The Food and Drug Administration
SC: Stratum corneum
IL-1ɑ: Interleukin-1 alpha
IL-1β: Interleukin-1 beta
TNF-ɑ: Tumor necrotic factor alpha
X-linked HGPRT: X-linked hypoxanthine-guanine phosphoribosyltransferase

## References

[1] Ogunwole OA (2012). Production of biodiesel from jatropha oil (curcas oil). Res J Chem Sci.

[2] Sukjit T, Punsuvon V (2013). Process optimization of crude palm oil biodiesel production by response surface methodology. Eur Int J Sci Technol.

[3] Johnson DT, Taconi KA (2007). The glycerol glut: options for the value-added conversion of crude glycerol resulting from biodiesel production. Environ Prog.

[4] Zhou CH, Beltramini JN, Fan YX, Lu GQ (2008). Chemoselective catalytic conversion of glycerol as a biorenewable source to valuable commodity chemicals. Chem Soc Rev.

[5] Chethan SG, Moinuddin-Khan MH, Sreepathi LK (2018). A novel method for refining crude glycerol a byproduct from biodiesel industries. J Applicable Chem.

[6] Hajek M, Skopal F (2010). Treatment of glycerol phase formed by biodiesel production. Bioresour Technol.

[7] Manosak R, Limpattayanate HS (2001). Sequential-refining of crude glycerol derived from waste used-oil methyl ester plant via a combined process of chemical and adsorption. Fuel Process Technol.

[8] Surrod S, Pattamaprom C (2011). Purification of glycerin by-product from biodiesel production using electrolysis process.

[9] Ueoka H, Katayama T (2001). Process for preparing glycerol.

[10] Gabriele C, Rutger AS (2007). Catalysis for renewables: from feedstock to energy production.

[11] Ampaitepin S, Tetsuo TA (2010). Perspective on incorporation of glycerin purification process in biodiesel plants using waste cooking oil as feedstock. Energy.

[12] Tan HW, AbdulAziz AR, Aroua MK (2013). Glycerol production and its applications as a raw material: a review.

[13] Stamatelatou K (2011). Advanced oil crop biorefineries.

[14] Erhirhie EO, Ihekwereme CP, Ilodigwe EE (2018). Advances in acute toxicity testing: strengths, weaknesses and regulatory acceptance. Interdiscip Toxicol.

[15] Lipnick RL, Cotruvo RN, Hill RD, Bruce KA, Stitzel AP, Walker I, Chu M, Goddard L, Segal JA, Myers RC (1995). Comparison of the up-and-down, conventional LD50 and fixed dose acute toxicity procedures. Fd Chern Toxicol.

[16] Parasuraman S (2011). Toxicological screening. J Pharmacol Pharmacother.

[17] Datson NA, van der Perk J, de Kloet ER, Vreugdenhil E (2001). Expression profile 30,000 genes in rat hippocampus using SAGE. Hippocampus.

[18] Venter JC, Adams MD, Myers EW (2001). The sequence of the human genome. Science.

[19] Gibbs RA, Weinstock GM, Metzker ML (2004). Genome sequence of the Brown Norway rat yields insights into mammalian evolution. Nature.

[20] Alessio HM, Ansinelli H, Threadgill C, Hagerman AE (2014) Comparison of gene and protein expressions in rats residing in standard cages with those having access to an exercise wheel. Biomed Res Int: ID 950516. PMCID: PMC3955688, PMID: 24719897. 10.1155/2014/950516 10.1155/2014/950516 PMC3955688 24719897

[21] Pelizaro BI, Braga FC, Crispim BA (2019). Assessment of acute toxicity and cytotoxicity of fluorescent markers produced by cardanol and glycerol, which are industrial waste, to different biological models. Environ Sci Pollut Res.

[22] Bremmer HJ, Prud'homme de Lodder LCH, van Engelen JGM (2006). Cosmetics Fact Sheet: To assess the risks for the consumer. Updated version for ConsExpo 4.

[23] Re-evaluation of glycerol (E 422) as a food additive. (2017). Available at: https://efsa.onlinelibrary.wiley.com/doi/full/10.2903/j.efsa.2017.4720.

[24] Hashish E, Elgaml S, El-fattah A, Shalaby S, Abdelaziz S (2020). β-Amyrin supplementation ameliorates the toxic effect of glycerol in the kidney of rat model. Hum Exp Toxicol.

[25] Hegazy AM, Hafez AS, Eid RE (2020). Protective and antioxidant effects of copper-nicotinate complex against glycerol-induced nephrotoxicity in rats. Drug Chem Toxicol.

[26] Mansouri MT, Hemmati AA, Bahareh Naghizadeh B, Mard SA, Rezaie A, Ghorbanzadeh B (2015). A study of the mechanisms underlying the anti-inflammatory effect of ellagic acid in carrageenan-induced paw edema in rats. Indian J Pharmacol.

[27] Fehrenbacher JC, Vasko MR, Duarte DB (2012). Models of inflammation: Carrageenan- or complete Freund’s Adjuvant (CFA)-induced edema and hypersensitivity in the rat. Curr Protoc Pharmacol.

[28] Gilligan JP, Lovato SJ, Erion MD, Jeng AY (1994). Modulation of carrageenan- induced hind paw edema by substance P. Inflammation.

[29] Halici Z, Dengiz GO, Odabasoglu F, Suleyman H, Cadirci E, Halici M (2007). Amiodarone has anti-inflammatory and anti-oxidative properties: An experimental study in rats with carrageenan-induced paw edema. Eur J Pharmacol.

[30] Fluhr JW, Darlenski R, Surber C (2008). Glycerol and the skin: holistic approach to its origin and functions. Br J Dermatol.

[31] Korponyai C, Szel E, Behany Z, Varga E, Mohos G, Dura A, Dikstein S, Kemeny L, Eros G (2017). Effects of locally applied glycerol and xylitol on the hydration, barrier function and morphological parameters of the skin. Acta Derm Venereol.

[32] Menegueti MG, Laus AM, Ciol MA, Martins MA, Filho AB, Gir E, Pires D, Pittet D, Rodrigues FB (2019). Glycerol content within the WHO ethanol-based handrub formulation: balancing tolerability with antimicrobial efficacy. Antimicrob Resist Infect Control.

[33] Atanda AT, Raphael S (2013). Role of surgeons in determining outcome of histopathology specimens. Niger J Surg.

[34] Panzuti P, Vidémont E, Fantini O (2020) A moisturizer formulated with glycerol and propylene glycol accelerates the recovery of skin barrier function after experimental disruption in dogs. Vet Dermatol. 10.1111/vde.12859 10.1111/vde.12859 PMC7586792 32628309

[35] Batt MD, Davis WB, Fairhurst E (1988). Changes in the physical properties of the stratum corneum following treatment with glycerol. J Soc Cosmet Chem.

[36] Nooshabadi VT, Khanmohamadi M, Valipour E et al (2020) Impact of exosome-loaded chitosan hydrogel in wound repair and layered dermal reconstitution in mice animal model. J Biomed Mater Res:1–12 10.1002/jbm.a.36959 32319166

[37] Fonseca CA, Pereira DG (2004). Aplicação da genetic toxicological em planta com atividade medicinal. Infarma.

[38] Rand MD (2010). Drosophotoxicology: the growing potential for Drosophila in neurotoxicology. Neurotoxicol Teratol.

[39] Rand MD, Montgomery SL, Prince L, Vorojeikina D (2015). Developmental toxicityassays using the *Drosophila* model. Curr Protoc Toxicol.

[40] Hifzur R, Siddique D, Kar Chowdhuri DK, Dhawan A (2005). Validation of *Drosophila melanogaster* as an *in vivo* model for genotoxicity assessment using modified alkaline Comet assay. Mutagenesis.

[41] Gaivão I, Sierra LM (2014). Drosophila comet assay: insights, uses, and future perspectives. Front Genet.

[42] Zemheri-Navruz F (2019). An optimized comet assay protocol for Drosophila Melanogaster. Bartın University. Int J Nat Appl Sci.

[43] Rajaei F, Karja N, Agung B, Wongsrikeao P, Taniguchi M, Murakami M, Sambuu R, Nii M, Otoi T (2005). Analysis of DNA fragmentation of porcine embryos exposed to cryoprotectants. Reprod Domest Anim.

[44] Yildiz C, Ottaviani P, Law N, Ayearst R, Liu L, McKerlie C (2007). Effects of cryopreservation on sperm quality, nuclear DNA integrity, in vitro fertilization, and in vitro embryo development in the mouse. Reproduction.

[45] Cunha JA, Feddern V, Marina C, Martha M, Higarashi, Paulo G, de Abreu, Coldebella A (2013) Synthesis and characterization of ethylic biodiesel from animal fat wastes. Fuel 105:228–234

[46] Chapter IV. Guidelines for Toxicity Tests - FDA. (2017). Available at: https://www.fda.gov/downloads/Food/GuidanceRegulation/GuidanceDocumentsRegulatoryInformation/IngredientsAdditivesGRASPackaging/UCM078734. Accessed 20 Nov 2019

[47] Semler DE (1992). The rat toxicology in animal models in toxicology.

[48] Lanhers CM, Fleurentin J, Mortier F, Vinche A, Younos C (1992). Anti-inflammatory and analgestic effects of aqueous extract of Harpagophtumprocumbens. Planta Med.

[49] Bancroft JD, Gamble M (2002). Theory and practice of histological techniques.

[50] Drury RAB, Wallington EA (1980). Preparation and fixation of tissues. Carleton’s histological technique. 5.

[51] Ashburner M, Golic KG, Hawley RS (2005). Drosophila: a laboratory handbook.

[52] Singh NP, McCoy MT, Tice RR, Schneider EL (1988). A simple technique for quantitation of low levels of DNA damage in individual cells. Exp Cell Res.

[53] Tice RR, Agurell E, Anderson D, Burlinson B, Hartmann A, Kobayashi H, Miyamae Y, Rojas E, Ryu JC, Sasaki YF (2000). Single cell gel/comet assay: guidelines for in vitro and in vivo genetic toxicology testing. Environ Mol Mutagen.

[54] Nanda MR, Yuan Z, Qin W, Poirier MA, Chunbao X (2014) Purification of crude glycerol using acidification: effects of acid types and product characterization. Austin Chem Eng 1(1):1004

[55] McEvoy GK (1993). American hospital formulary service - drug information.

[56] Gosselin RE, Smith RP, Hodge HC (1984). Clinical toxicology of commercial products.

[57] Zaman RT, Parthasarathy AB, Vargas G, Chen B, Dunn AK, Rylander HG, Welch AJ (2009). Perfusion in hamster skin treated with glycerol. Lasers Surg Med.

[58] Szél E, Polyánka H, Szabó K (2015). Anti-irritant and anti-inflammatory effects of glycerol and xylitol in sodium lauryl sulphate-induced acute irritation. J Eur Acad Dermatol Venereol.

[59] Gittler JK, Krueger JG, Guttman-Yassky E (2013). Atopic dermatitis results in intrinsic barrier and immune abnormalities: implications for contact dermatitis. J Allergy Clin Immunol.

[60] Cumberbatch M, Bhushan M, Dearman RJ, Kimber I, Griffiths CEM (2003). IL-1β-induced Langerhans’ cell migration and TNF-α production in human skin: regulation by lactoferrin. Clin Exp Immunol.

[61] Elwell MR, Stedman MA, Kovatch RM (1990). Skin and subcutis. Pathology of the Fischer rat: reference and atlas.

[62] Klein-Szanto AJP, Conti CJ (2002). Skin and oral mucosa. Handbook of toxicologic pathology.

[63] Peckham JC, Heider K (1999). Skin and subcutis. Pathology of the mouse: reference and atlas.

[64] Yousef H, Alhajj M, Sharma S (2020). Anatomy, skin (integument), epidermis. StatPearls.

[65] Batt MD, Fairhurst E (1986). Hydration of the startum corneum. J Soc Cosmet Chem.

[66] Björklund S, Engblom J, Thuresson K, Sparr E (2013). Glycerol and urea can be used to increase skin permeability in reduced hydration conditions. Eur J Pharm Sci.

[67] Okamoto T, Inoue H, Anzai S (1998). Skin-moisturizing effect of polyols and their absorption into human stratum corneum. Int J Cosmet Sci.

[68] Warren B, Shapiro WR (1996). Glycerin moisturizers. A supplement to cosmetic dermatology.

[69] Doolittle DJ, Lee DA, Lee CK (1988). The genotoxic activity of glycerol in an in vitro test battery. Int J Food Food Toxicol.

